# Association between thyroid stimulating hormone levels and papillary thyroid cancer risk: A meta-analysis

**DOI:** 10.1515/biol-2022-0671

**Published:** 2023-08-11

**Authors:** Bin Xu, Shu-Yan Gu, Ning-Ming Zhou, Jun-Jie Jiang

**Affiliations:** Department of Ultrasound, Shanghai Fifth People’s Hospital, Fudan University, 128 Ruili Road, Minhang District, Shanghai, 200240, China

**Keywords:** thyroid stimulating hormone, papillary thyroid cancer, meta-analysis

## Abstract

High thyroid stimulating hormone (TSH) levels may stimulate papillary thyroid cancer (PTC) cell proliferation; however, the relationship between TSH levels and PTC risk remains controversial. We aim to ascertain the association through a meta-analysis. Literature searches were conducted in PubMed, Embase, and Web of Science databases. After literature screening, the methodological quality was assessed using the Newcastle-Ottawa Scale and Agency for Healthcare Research and Quality methods. Cochran’s *Q* and *I* ^2^ tests were used to evaluate heterogeneity in the meta-analysis. Egger’s test was applied to assess publication bias. A total of 12 eligible studies were included in this meta-analysis; all were of moderate and high methodological quality. The pooled results suggested that increased TSH levels were significantly associated with PTC risk; however, the included studies were significantly heterogeneous. Stratification analysis indicated that the heterogeneity might be from the area or type of control. Although significant publication bias existed among the studies, the trim-and-fill method and sensitivity analysis revealed that the combined results were stable and robust. TSH levels are significantly associated with the PTC risk; however, more high-quality studies in large sample sizes are recommended to verify the extrapolation of these findings.

## Graphical abstract

**Figure:**
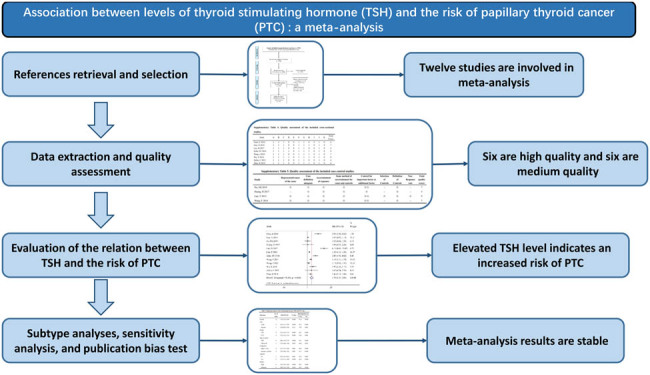

## 1 Introduction

Thyroid cancer is the most prevalent of all endocrine malignancies worldwide, and its rapid increase in incidence can be attributed to papillary thyroid cancer (PTC) [1,2]. As the most widespread histological subtype of thyroid cancer, PTC accounts for 80–90%, but with a superior prognosis than other subtypes [3,4]. The 10-year survival rate of PTC is estimated to be approximately 93%; however, 25–35% of patients still exhibit tumor heterogeneity as well as aggressive variants with unique clinical, pathological, and molecular characteristics [5,6]. The current recommendation is to use ultrasound (US) and fine-needle aspiration cytology (FNAC) to diagnose malignancy, along with surgical treatment as first-line therapy [7]. Although FNAC is the gold standard for assessing thyroid nodules, it still carries the risk of trauma and of causing the spread of tumor cells and has approximately 10% diagnostic uncertainty [8]. Clinical guidelines also suggest incorporating specific clinicopathological features, such as age, sex, tumor size, and *BRAF* mutations, to reduce overdiagnosis and indicate the probability of recurrence [9,10]. Therefore, there is a need to discover new molecular markers and apply them to the diagnosis and treatment of PTC to better predict the malignancy and aggressiveness of the lesions.

Thyroid stimulating hormone (TSH) is a pituitary hormone whose serum or plasma levels are a sensitive marker for assessing hyper- or hypothyroidism [11]. TSH is regulated by the negative feedback from triiodothyronine and thyroxine and is influenced by stress hormones and cytokines during homeostasis [12]. In addition to genetic factors, demographic factors (such as sex and age), intrinsic factors (such as microbial community and stress response), drug use, and environmental factors, all contribute to inter-individual variation in TSH levels [13]. TSH suppression is now considered to significantly reduce the postoperative recurrence risk of low-risk differentiated thyroid cancer [14]. A higher TSH level may also activate cell proliferation and thyroglobulin production in PTC [15]. However, there is still controversy about the relationship between TSH levels and the risk of developing PTC. It has been suggested that a significant positive correlation exists between the risk of PTC and TSH levels, particularly in cases of PTC with tumor sizes less than 1.0 cm [16]. However, another case–control study found a negative correlation between PTC risk and TSH levels within the normal range [17].

Therefore, this meta-analysis aimed to summarize observational studies on TSH levels and PTC risks and comprehensively assess and explore their association. This study’s findings will provide evidence for prediagnostic risk assessment of PTC to reduce invasive testing and overdiagnosis among patients.

## 2 Methods

### 2.1 Literature retrieval

Literature retrieval was carried out through PubMed, Embase, and Web of Science databases. Search keywords included “thyrotropin,” “thyrotropic hormone,” “thyroid stimulating hormone,” and “papillary thyroid cancer.” Keywords in the same category were joined together with “OR,” while keywords in different categories were joined together with “AND.” The search process combined subject terms with free terms, and the search strategy was adapted to the characteristics of the database. Detailed retrieval procedures of each database are displayed in Tables S1–S3. The search was available until January 12, 2023, without language restrictions. This study also screened relevant reviews and their included literature to expand the search scope of this meta-analysis.

### 2.2 Inclusion and exclusion criteria of literature screening

Inclusion criteria: (1) the study reports TSH and PTC incidence risk, (2) the study type is cross-sectional, case–control, or cohort, and (3) the odds ratio (OR) and 95% confidence interval (CI) with univariate or multivariable adjustment are reported, or can be calculated by converting data based on frequency, sample size, etc.

Exclusion criteria: (1) reviews, conference abstracts, and comments, (2) studies with participants under 18 years of age, and (3) for duplicate publications or multiple papers with the same batch of data, only the study with the most comprehensive data was kept, and the rest were excluded.

### 2.3 Data extraction and quality evaluation

Two independent researchers conducted literature screening and data extraction based on the above criteria. Information to be extracted includes first author, publication year, region where the study was conducted, type of study, basic characteristics of the study population (sample size, gender, and age), TSH grouping, study outcomes, and correction factors. Any inconsistency in data extraction will be solved after discussion. The methodological quality of the case–control and cohort studies was appraised according to the Newcastle-Ottawa Scale (NOS) [18], which included three aspects of selection, comparability, and exposure, with a total of eight scoring items and each of which is scored out of nine. Of these, scores of 7–9, 4–5, and 0–3 were assessed as high-, moderate-, and low-quality studies, respectively. The methodological quality of the cross-sectional study was evaluated by the Agency for Healthcare Research and Quality (AHRQ) [19]. A total of 11 entries were assessed using “yes,” “no,” and “unclear,” with “yes” scoring 1 while “no” and “unclear” scoring 0. For these, scores of 8–11, 4–7, and 0–3 were considered as high-, moderate-, and low-quality studies, respectively.

### 2.4 Statistical analysis

To assess the relationship between TSH and the risk of PTC, the OR and 95% CI were adopted as effect size indicators. If the included studies classified TSH into multiple categories, such as <0.40, 0.40–1.35, 1.36–2.12, 2.13–4.20, and >4.20 μIU/mL, then TSH <0.40 μIU/mL was used as the control group, and the OR (95% CI) of the remaining subgroups was combined in a random-effects model to calculate OR (95% CI) of ≥0.4 vs <0.40 μU/mL (high vs low) for the following meta-analysis. If the included studies used TSH as a continuous variable to calculate the risk of PTC occurrence, then this study used an increase of 1 μIU/mL as the dose change value for TSH and calculated its corresponding OR (95% CI) for the following meta-analysis.

Cochran’s *Q* test and *I* ^2^ test [20] were then performed to estimate the heterogeneity among the studies. The significant heterogeneity was defined at *P* < 0.05 or *I* ^2^ > 50%, and the random-effects model was therefore applied for the meta-analysis. Otherwise, the fixed effect model was adopted instead. Subgroup analysis was performed according to geographical location, study type, type of control, TSH grouping type, confounding factors adjusted or not, and methodological quality. A one-by-one elimination test [21] was used to determine whether the effect of the individual included study on the results of the meta-analysis was significant. Egger’s test [22] was used to evaluate publication bias. When significant publication bias existed, the trim-and-fill method [23] was employed to evaluate the stability of the combined results. The above statistical analyses were generated using Stata 12.0 software.

## 3 Results

### 3.1 Literature search and screening

**Figure 1:**
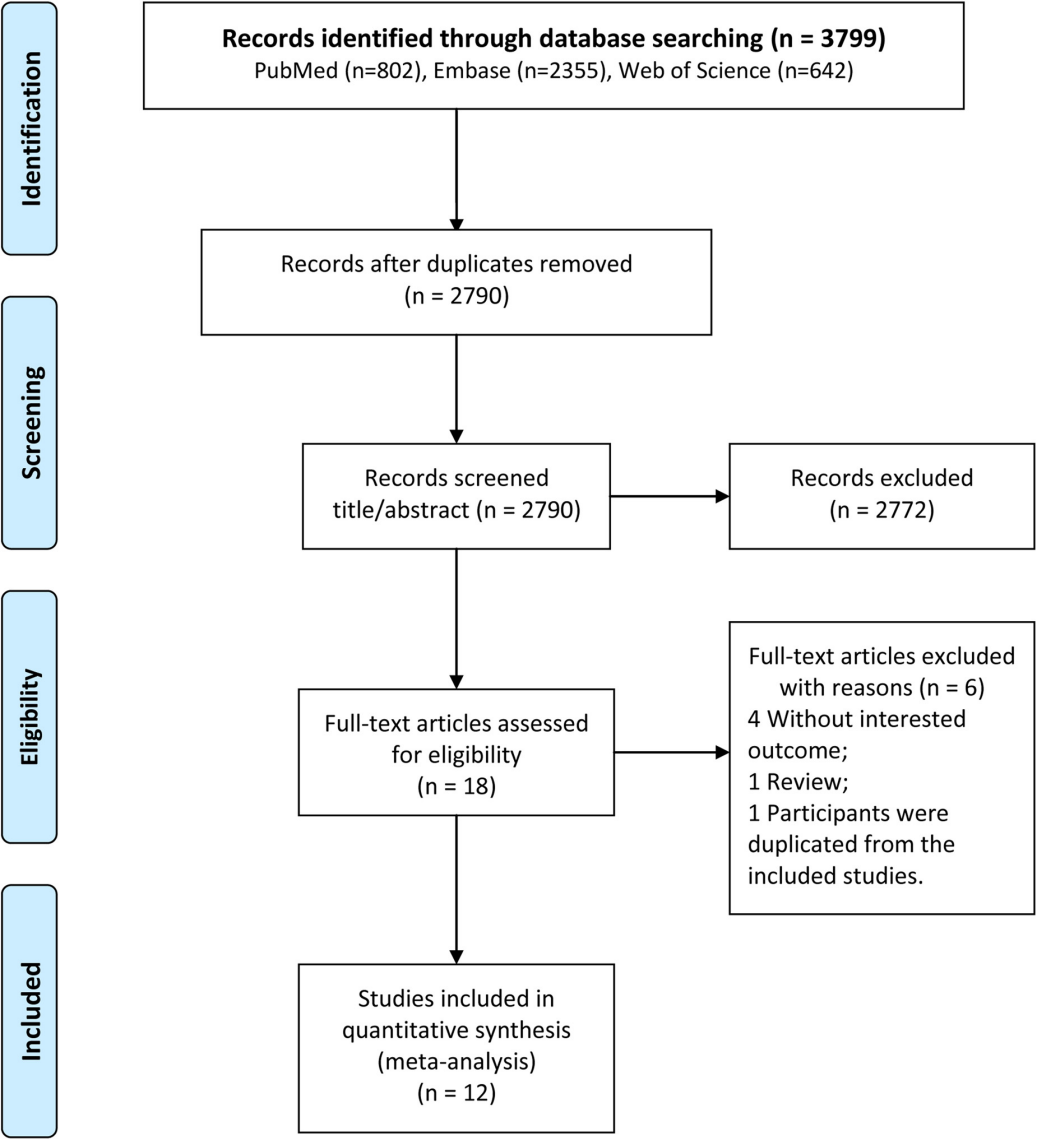
Detailed literature retrieval process and results in this meta-analysis.

The detailed retrieval process and results are shown in Figure 1. Based on three databases, 3,799 records were obtained. After excluding 1,009 duplicates, 2,790 records remained. Subsequently, 2,772 articles that did not meet the inclusion criteria were excluded after browsing the titles and abstracts. Furthermore, six articles were eliminated after full-text assessment and the remaining eligible 12 studies [16,17,24–33] were finally included in this meta-analysis.

### 3.2 Characteristics and methodological quality evaluation of the included studies

As shown in Table 1, the 12 included studies were published between 2010 and 2022 and were conducted in China, Korea, Poland, the USA, Spain, and other regions. Of these, eight were cross-sectional [24–27,29,31–33], and four were case–control studies [16,17,28,30]. The sample size for inclusion in the study ranged from 233 to 27,914, with 7,774 patients with PTC and 33,909 controls enrolled in this meta-analysis. Other detailed information, such as the study participants’ age, study outcomes, and correction factors, are exhibited in Table 1.

**Table 1:** Characteristics of 12 included studies in this meta-analysis

Study	Location	Design	*n*, M/F	Case/control	Age (years)	Type of control	Categories of TSH (μU/mL)	OR (95% CI)	Adjusted factors
Fiore et al. [25]	Italy	CSS	27,914, 5,249/22,665	1,275/26,639	40.0 ± 12.9	TBP	≥0.4 vs <0.4	3.862 (2.476, 6.024)	None
Guo et al. [26]	China	CSS	258, 62/196	153/105	45.7 ± 11.6/48.4 ± 11.9#	TBP	Per 1 μIU/mL	1.054 (0.972, 1.142)	Fasting insulin, HOMA-IR, TPOAb
Hu et al. [16]	China	CCS	649, 208/441	320/329	47.5 ± 15.0	TBP, HC	≥1.32 vs <1.32	1.021 (0.693, 1.503)	Sex, age, income, BMI, physical activity, history of CT scan or thyroid diseases, diabetes, fasting serum glucose, and urinary iodine/creatinine ratio
Huang et al. [17]	USA	CCS	1,482, 800/682	741/741	NR	TBP, HC	>1.93 vs 1.20–1.93	1.060 (0.512, 2.196)	BMI and branch of military service
Lee et al. [27]	Taiwan	CSS	368, 58/310	127/241	47.7 ± 12.7/46.1 ± 14.7#	TBP	0.14–4.52 vs <0.14	8.113 (4.886, 13.472)	None
Lun et al. [28]	China	CCS	2,478, 524/1,954	676/1,802	44.2 ± 13.2/49.4 ± 11.3#	TBP	Per 1 μIU/mL	1.28 (1.19, 1.38)	HT, sex, age, T3, T4, TGAB, and TPOAB
Sohn et al. [29]	Korea	CSS	3,791, 597/3,194	2,881/910	46.8 ± 11.8/44.1 ± 13.0#	TBP	≥0.4 vs <0.40	2.802 (1.954, 4.016)	Sex, age, solitary nodule, HT
Wang et al. [30]	China	CCS	460, 111/349	103/357	50.5 ± 10.3/49.5 ± 11.2#	TBP, HC	Per 1 μIU/mL	1.53 (1.27, 1.70)	FT3, FT4, TgAb, TPOAb, age, sex, urine iodine, and thyroid nodule size
Wang et al. [24]	China	CSS	233, 58/175	81/152	44 (37, 54)	TBP, HC	Per 1 μIU/mL	1.145 (0.928, 1.413)	T3, T4, TGAb, TPOAb, NDRG3
Wu et al. [31]	China	CSS	2,132, 482/1,650	537/1,595	45.0 ± 11.6/49.3 ± 13.4#	TBP	≥0.35 vs <0.35	1.972 (1.251, 3.108)	FT3, FT4, TAb, TgAb, TPOAb, age, sex, thyroid nodules
Zafon et al. [32]	Spain	CSS	346, NR	36/310	52.1 ± 14.7/54.4 ± 14.2#	TBP	>4.0 vs <0.40	1.670 (0.742, 3.759)	None
Zhao et al. [33]	China	CSS	1,572, 0/1,572	844/728	45.1 ± 10.6/49.7 ± 11.8#	TBP	>1.697 vs <1.697	1.46 (1.13, 1.90)	Age, TPOAb, TGAb, urinary iodine, nodule size and number

TBP, thyroid benign patients; HC, healthy control; CSS, cross-sectional study; CCS, case–control study; T3, triiodothyronine; T4, tetraiodothyronine; TAb, thyroid autoantibodies; TGAb, thyroglobulin antibody; TPOAb, thyroidperoxidase antibody; TSH, thyrotropin; HOMA-IR, homeostasis model assessment of insulin resistance; HT, Hashimoto’s thyroiditis; BMI, body mass index; NR, not reported; M, male; F, female. #, case/control.

The results of the quality assessment suggested AHRQ scores of 6–8 for cross-sectional studies and NOS scores of 5–9 for case–control studies (Tables S4 and S5). Thus, six of the 12 included articles are high-quality studies [16,28–31,33], and the remaining six [17,24–27,32] are of medium methodological quality, with the main types of bias being recall and confounding bias.

### 3.3 Meta-analysis

**Figure 2:**
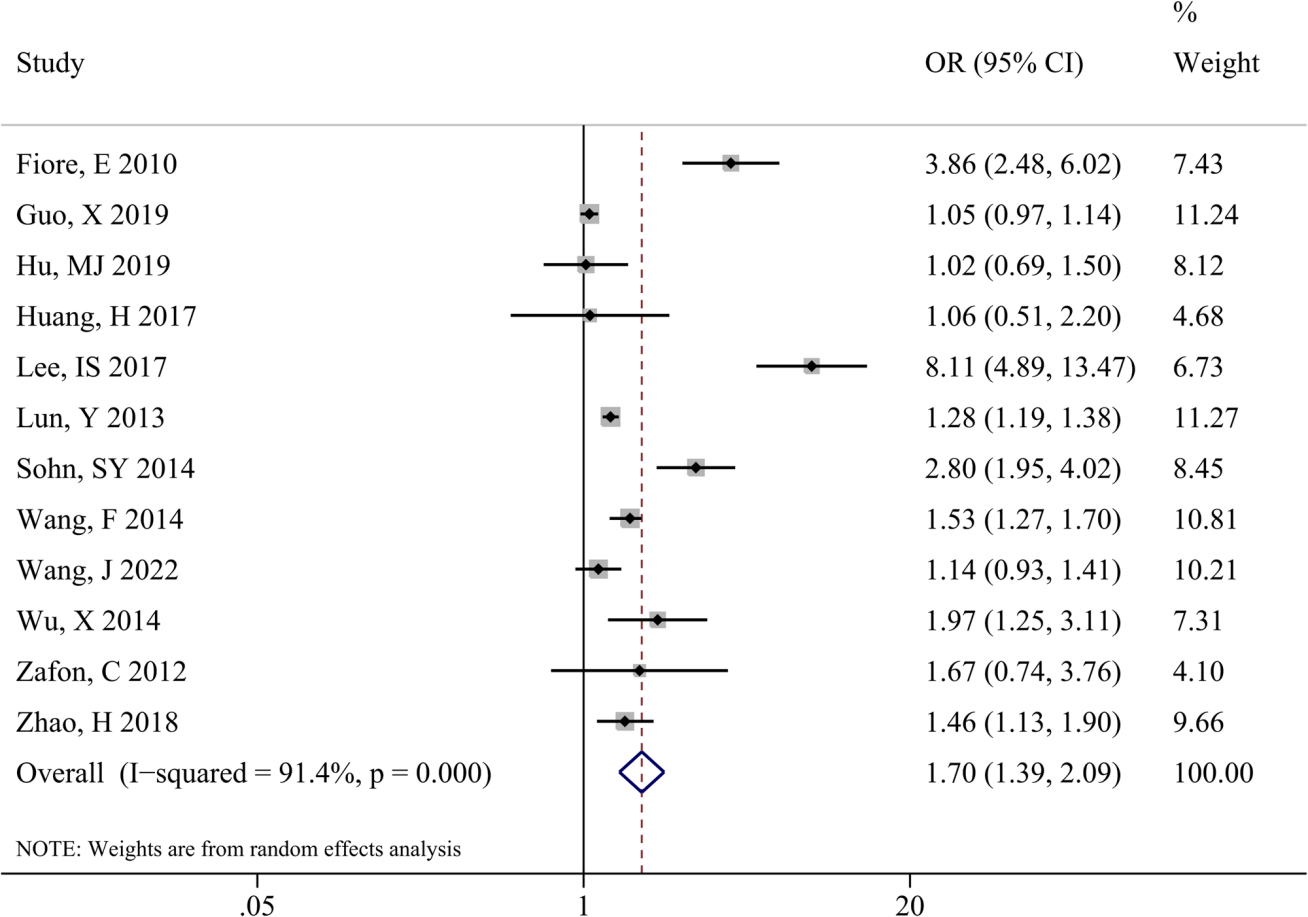
Forest plot depicts the combined results of 12 included studies on the association between TSH levels and PTC risk.

These 12 studies were incorporated in the meta-analysis to appraise the relationship between TSH and the onset risk of PTC. The results indicated a significant heterogeneity between studies at *I* ^2^ = 91.4% and *P* < 0.001, and the random-effects model was used in this meta-analysis. The pooled results estimated a significant association between elevated TSH levels and an increased risk of PTC, with OR (95% CI) = 1.70 (1.39, 2.09) and *P* < 0.001 (Figure 2).

### 3.4 Subgroup analysis

The subgroup analysis was conducted to identify sources of heterogeneity further (Table 2). After stratifying the included studies according to geographical location, study type, control type, TSH variable type, confounders adjusted or not, and methodological quality, all subgroups still suggested significant heterogeneities (*I* ^2^ > 50%), indicating that none of the grouping factors mentioned above contributed to the heterogeneity of combined results. In addition, except for the Western area and the control type of thyroid benign patients (TBP) or healthy control (HC), the results for all subgroups were consistent with the combined results, suggesting that elevated TSH levels may increase the risk of PTC (OR > 1, *P* < 0.01).

**Table 2:** Subgroup analyses of the relationship between TSH and PTC risk

Outcomes	No. of study	OR (95%CI)	*P* value	Heterogeneity test	
				*I* ^2^ (%)	*P*_H_
Overall	12	1.70 (1.39, 2.09)	<0.001	91.4	<0.001
Area					
Asian	9	1.61 (1.31, 1.99)	<0.001	92.3	<0.001
Western	3	1.98 (0.85, 4.59)	0.111	79.9	0.007
Design					
CSS	8	2.12 (1.42, 3.17)	<0.001	94.1	<0.001
CCS	4	1.32 (1.14, 1.53)	<0.001	54.8	0.085
Type of control					
TBP	8	2.08 (1.56, 2.77)	<0.001	94.1	<0.001
TBP or HC	4	1.25 (1.00, 1.56)	0.055	60.6	0.055
Comparison					
High vs Low	8	2.17 (1.37, 3.44)	0.001	88.6	<0.001
Increase 1 μIU/mL	4	1.24 (1.06, 1.45)	0.007	87.3	<0.001
Adjusted					
No	3	3.95 (1.81, 8.61)	0.001	82.4	0.003
Yes	9	1.37 (1.17, 1.60)	<0.001	84.4	<0.001
Quality					
High	6	1.54 (1.26, 1.88)	<0.001	80.4	<0.001
Moderate	6	2.00 (1.18, 3.39)	0.010	94.5	<0.001

TSH, thyrotropin; PTC, papillary thyroid cancer; OR, odds ratio; CI, confidence interval; CSS, cross-sectional study; CCS, case–control study; TBP, thyroid benign patients; HC, healthy control.

### 3.5 Sensitivity analysis and publication bias test

**Figure 3:**
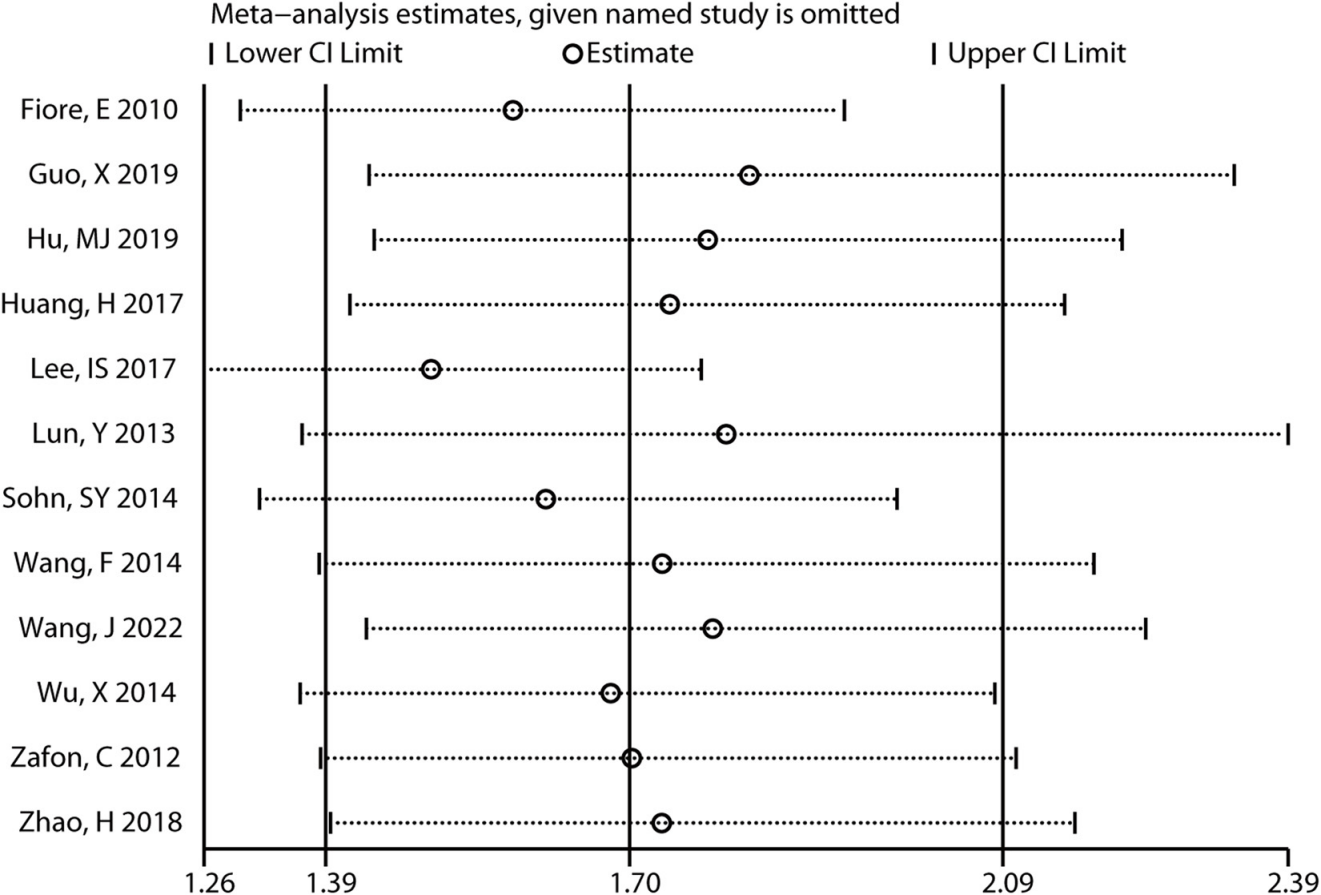
Sensitivity analysis displays the change in the combined results after removing individual studies one by one.

Sensitivity analysis showed a change in OR (95% CI) from 1.50 (1.26, 1.78) to 1.83 (1.43, 2.33) for the combined results after eliminating articles one by one (Figure 3). Meanwhile, after excluding any single study, the combined results of the remaining studies maintained statistical significance at *P* < 0.05, suggesting that the original pooled result was stable and not significantly altered by individual studies.

Furthermore, Egger’s test was conducted to ascertain publication bias, and the result suggested a significant publication bias at *P* = 0.039 among the included studies. However, with the trim-and-fill method, the procedure did not fill in the dummy negative results to enhance the symmetry of the funnel plot, indicating that the meta-analysis results were stable.

## 4 Discussion

A recent study found that sensitivity to thyroid hormone indices was an indicator for predicting PTC [34]. TSH is the thyroid gland’s main regulator and growth factor, and its level is somewhat indicative of thyroid function. TSH inhibition is reported to inhibit the cell proliferation of thyroid cancer and reduce disease progression and recurrence, thereby improving the survival status of patients with PTC [35]. However, studies are inconsistent on the relationship between TSH levels and PTC risk. We integrated data from 12 moderate- and high-quality observational studies and performed this meta-analysis. The combined results suggested that there may be an observable association between elevated TSH levels and an increased risk of developing PTC. A relevant meta-analysis confirmed our results and suggested a prominent relation between higher TSH levels and the risk of differentiated thyroid cancer [36]. In addition, each 1 mU/L increase in serum TSH was correlated with a 14% increase in the risk of all histological types of thyroid cancer and a 22% increase in the risk of PTC [37]. Therefore, depending on the US features and size, we recommend that FNAC be carried out on nodules in patients with high TSH levels to avoid invasive testing and overdiagnosis. Finally, preoperative assessment in patients can affect cancer postoperative outcomes and overall survival [38]. TSH levels also demonstrated the ability to risk of extrathyroidal spread and central lymph node metastasis [39].

Although the above evidence suggests that the increased serum TSH level is correlated with cancer risk in thyroid nodules, the basis of this view is that TSH affects the proliferation of malignant transformed cells of differentiated thyroid cancers and thyroid cells [39]. The stratified results of this study suggested that the association between TSH levels and PTC risks was not significant in the subgroup where the controls contained the healthy population. These findings indicated that changes in TSH levels might only be of significance in the progression of benign nodules to thyroid cancer. The possible reason is that TSH receptor expression is increased in benign adenomas and thyroid cancers compared to normal thyroid tissues, and TSH binding to its receptor promotes cell development and proliferation, as well as PTC progression [40]. Therefore, we recommend using TSH level to predict the PTC onset risk among patients with benign thyroid nodules. In addition, the subgroup analysis in this study also revealed that the correlation between TSH levels and PTC risk is significant in Asian countries but not in Western countries. Although TSH is a sensitive and specific biomarker of thyroid status, age, sex, ethnicity, iodine status, body mass index (BMI), smoking, and other factors can all affect TSH levels [41]. For example, TSH levels increase with body weight and are positively correlated with BMI levels [42,43]. However, obesity rates in high-income countries in Western regions such as North America, Oceania, and Europe exceed those in Asia [44]. BMI also varies significantly across ethnic groups, with Chinese, Koreans, and Vietnamese having lower BMIs than Whites [45]. Furthermore, cigarette smoking causes a decrease in TSH levels, possibly due to elevated serum FT4 and FT3 levels caused by sympathetic nervous system activation [46]. The current data support that European smokers have more tobacco consumption per day and therefore have higher cumulative pack-years than South Asians [47]. Meanwhile, excess iodine can lead to excessive TSH synthesis and release, as well as decreased thyroid hormone levels [48]. The global data suggest that excess iodine intake in school-age children is more prevalent in non-Asian regions [49]. Combined with the results of this study, it is suggested that additional attention needs to be paid to variables and factors such as BMI, smoking status, and iodine intake that significantly affect TSH levels when assessing the correlation between TSH levels and PTC risk in Western populations.

The highlights of this study are as follows: (1) The effect values of this meta-analysis results were relatively large, suggesting a high strength of association between TSH levels and PTC risk. (2) The combined results do not alter significantly under the influence of any individual study, and the results are therefore robust. (3) Although the included studies exhibited significant publication bias, the trim-and-fill method suggested that the pooled results were stable. However, this study has some limitations: (1) The included studies were significantly heterogeneous, and subgroup analysis did not clarify the source of heterogeneity. (2) All included studies are observational studies with numerous confounding factors. Although most studies have conducted multi-factor correction, the corrected factors are not uniform, which may exaggerate the correlation strength between TSH and PTC. (3) The inconsistent grouping criteria of TSH levels may lead to clinical and statistical heterogeneity. It is hoped that follow-up studies can determine the uniform grouping criteria.

## 5 Conclusion

The combined results of this meta-analysis suggested a significant association between high levels of TSH and elevated risk of PTC. However, this relationship still needs to be supported by more evidence in the Western subgroup, as well as in the subgroup of controls containing healthy populations. Furthermore, more high-quality studies with larger sample sizes are recommended to validate the extrapolation of these findings.

## Supplementary Material

Supplementary Table
