# Supplementary material for: Association between thyroid stimulating hormone levels and papillary thyroid cancer risk: A meta-analysis
Source: Open Life Sci. 2023 Aug 11;18(1):20220671. doi: 10.1515/biol-2022-0671 (PMC10426723; doi:10.1515/biol-2022-0671)
Supplement: Supplementary Table [file biol-2022-0671-sm.pdf]

Supplementary material

Table S1: Retrieve procedures and results in PubMed

Search	Query	Items found
#1	("thyrotropin"[MeSH Terms] OR "thyrotropin"[All Fields] OR "thyrotropine"[All Fields] OR "thyrotropins"[All Fields]) OR "thyrotropic hormone"[All Fields] OR "hormothyrin"[All Fields] OR "thyroid stimulating hormone"[All Fields] OR "TSH"[All Fields]	64,954
#2	"thyroid cancer, papillary"[MeSH Terms] OR "papillary thyroid cancer"[All Fields] OR "papillary thyroid carcinoma"[All Fields]	14,396
#3	#1 AND #2	802

Table S2: Retrieve procedures and results in Embase

Search	Query	Items found
#1	('thyrotropin'/exp OR thyrotropin:ti,ab OR 'thyrotropine'/exp OR thyrotropine:ti,ab OR 'thyrotropic hormone'/exp OR 'thyrotropic hormone':ti,ab OR hormothyrin:ti,ab OR 'thyroid stimulating hormone'/exp OR 'thyroid stimulating hormone':ti,ab)	80,689
#2	('papillary thyroid cancer'/exp OR 'papillary thyroid cancer':ti,ab OR 'papillary thyroid carcinoma'/exp OR 'papillary thyroid carcinoma':ti,ab)	24,718
#3	#1 AND #2	2,355

Table S3: Retrieve procedures and results in Web of Science

Search	Query	Items found
#1	thyrotropin OR thyrotropine OR “thyrotropic hormone” OR hormothyrin OR “thyroid stimulating hormone” OR TSH (All Fields)	29,267
#2	"papillary thyroid cancer" OR "papillary thyroid carcinoma" (All Fields)	12,883
#3	#1 AND #2	642

Table S4: Quality assessment of the included cross-sectional studies

Study	A	B	C	D	E	F	G	H	I	J	K	Total Scores
Fiore et al. [25]	1	1	1	0	0	1	1	0	0	1	0	6
Guo et al. [26]	1	1	1	0	0	1	1	1	0	1	0	7
Lee et al. [27]	1	1	1	0	0	1	1	0	0	1	0	6
Sohn et al. [29]	1	1	1	1	0	1	1	1	0	1	0	8
Wang et al. [24]	1	1	1	0	0	1	1	1	0	1	0	7
Wu et al. [31]	1	1	1	1	0	1	1	1	0	1	0	8
Zafon et al. [32]	1	1	1	0	0	1	1	0	0	1	0	6
Zhao et al. [33]	1	1	1	0	0	1	1	1	0	1	0	8

A: Define the source of information (survey, record review); B: List inclusion and exclusion criteria for exposed and unexposed subjects (cases and controls) or refer to previous publications; C: Indicate time period used for identifying patients; D: Indicate whether or not subjects were consecutive if not population-based; E: Indicate if evaluators of subjective components of study were masked to other aspects of the status of the participants; F: Describe any assessments undertaken for quality assurance purposes (e.g., test/retest of primary outcome measurements); G: Explain any patient exclusions from analysis; H:Describe how confounding was assessed and/or controlled; I: If applicable, explain how missing data were handled in the analysis; J: Summarize patient response rates and completeness of data collection; K: Clarify what follow-up, if any, was expected and the percentage of patients for which incomplete data or follow-up was obtained.

Table S5: Quality assessment of the included case-control studies

Study	Representativeness of the cases	Case definition adequate	Ascertainment of exposure	Same method of ascertainment for cases and controls	Control for important factor or additional factor	Selection of Controls	Definition of Controls	Non-Response rate	Total quality scores
Hu et al. [16]	☆	☆	☆	☆	☆☆	—	☆	—	7
Huang et al. [17]	—	☆	☆	☆	☆	—	☆	—	5
Lun et al. [28]	☆	☆	☆	☆	☆☆	☆	☆	☆	9
Wang et al. [30]	☆	☆	☆	☆	☆☆	—	☆	—	7
